# Understanding Surgeons’ Reluctance to Adopt Intraoperative Coronary Graft Verification Procedures: A Literature Review Combined to AI-Driven Insights Under Human Supervision

**DOI:** 10.3390/jcm13226889

**Published:** 2024-11-15

**Authors:** Gabriele Di Giammarco, Federico Cammertoni, Nicola Testa, Massimo Massetti

**Affiliations:** 1 Department of Neuroscience, Imaging and Clinical Science, School of Medicine and Health Science, Università “G.D’Annunzio” Chieti–Pescara, 66100 Chieti, Italy; 2 Faculty of Medicine and Surgery, Catholic University of Sacred Heart, 00168 Rome, Italy; massimo.massetti@unicatt.it; 3 Department of Cardiovascular Sciences, Fondazione Policlinico Universitario “A. Gemelli” IRCCS, 00168 Rome, Italy; federico.cammertoni@policlinicogemelli.it (F.C.); ntesta.cch@gmail.com (N.T.)

**Keywords:** coronary surgery, coronary artery disease, coronary artery bypass grafting, intraoperative graft verification, transit-time flow measurement, high-resolution ultrasound, high-frequency ultrasound

## Abstract

**Background:** Intraoperative graft verification in coronary surgery is accepted worldwidand equally discussed. In spite of multiple sources of evidence published up to now in favor of clinical benefits following the use of the procedure, there is a persistent skepticism in adopting the available technologies. The object of the present review is to analyze the reluctance of surgeons toward the adoption of assessment methods. **Materials and Method:** A thorough literature review was carried out on Google Scholar based on the results obtained from AI’s answer to the question about the reasons for that reluctance. We took advantage of using ChatGPT-4 since the research based on PubMed Central alone was not able to return a detailed response, maybe because the reasons for the reluctance are veiled in the text of the published papers. Through the items suggested by AI and taken from the literature, we deepened the research, pointing attention to the issues published so far about the various technologies. **Results:** There are many convincing pieces of evidence about the utility of intraoperative graft control in coronary surgery, involving improved clinical outcome, efficacy and safety, and social cost saving. The opinion that arose through this analysis is that, beyond the objective difficulties in utilizing some technologies and the equally objective limitations of an economic and organizational nature, the reluctance is the result of a real unwillingness based on the various implications that the discovery of the technical error entails. **Conclusions:** This negative attitude, in light of the convincing scientific and clinical evidence published up to now, appears to overwhelm the benefits for patients.

## 1. Forewords

In recent years, the use of **generative artificial intelligence** (GAI) to assist in the creation of scientific manuscripts has gained traction. AI tools like GPT-3 and GPT-4 can enhance writing by summarizing large amounts of research, generating content drafts, and even providing grammar corrections. However, concerns about the ethical use of AI in academic writing persist. The European Committee for AI use emphasizes that AI should be used as an assistive tool rather than a substitute for humans [1].

Researchers must ensure transparency and proper attribution when AI is involved in the writing process [2].

AI-based writing tools bring several advantages but need to be carefully considered:

Efficiency: AI can reduce the time spent on drafting sections such as literature reviews, allowing researchers to focus on data analysis and critical thinking;

Clarity: for non-native English speakers, AI improves the clarity of scientific texts, making research more accessible to international audiences [3].

However, there are also drawbacks to consider:

Ethical concerns: over-reliance on AI raises issues of intellectual property and accountability, as AI-generated content might obscure the original contributions of the authors [4];

Bias: AI tools trained on biased data may perpetuate inaccuracies, leading to flawed conclusions in scientific manuscripts even if there are reports indicating that the Generative Artificial Intelligence can be trained to become more accurate [5].

This review was assisted by AI tool adhering to the European Ethical Guidelines cited above [1], with all contents generated having been critically reviewed by human supervision. It is clear that AI was not considered as an Au-thor bit just an informed assistant [6].

AI methodology and verification: as far as use of AI is concerned, we decided to go this route after unsuccessfully trying to search for the answer to the research question using the whole phrase or pertinent PubMed keywords. The attempts did not generate any reference. We therefore simply reported the question to ChatGPT 4o, “feeding” it with the result of the search on “intraoperative coronary grafts verification” previously performed on PubMed which returned more than 250 publications. The AI query reported in the title of the present manuscript identified the eight reasons hindering wide adoption. We afterwards searched for the publications by entering each reason in google scholar and checking for each bibliographic reference for the congruence of the citation.

Therefore, one must not overlook the limitations of these tools, which are fundamentally based on the understanding of context, which is particularly difficult for such systems when writing scientific texts. The nuances of context are an example of how such systems must be guided. It is not difficult to realize when an AI resource goes off the rails while maintaining the logical thread of context; this is why we have closely monitored all the phases for which we have made use of the collaboration of these systems.

## 2. Introduction

Coronary artery bypass grafting (CABG) is a critical intervention for patients suffering from coronary artery disease (CAD) [7,8,9,10,11,12]. Ensuring the patency of grafts during surgery is essential to avoid complications such as perioperative myocardial infarction and graft failure [13]. Traditionally, surgeons have relied on visual and manual methods to assess graft function, but these techniques can be subjective and often miss subtle issues [14]. Very surprisingly, surgeons are not so favorable towards using a method for graft function assessment [15] for different reasons. We hereafter try to analyze the reasons to provide some answers that hopefully would convince those skeptical minds.

## 3. Material and Methods

### 3.1. Intraoperative Graft Verification Technologies

Several methods have been developed to provide real-time verification of graft patency, with varying degrees of accuracy [16], invasiveness [17], and cost-effectiveness [18]. The most commonly used methods include the following:

***i.***  ***Transit-Time Flow Measurement (TTFM)***

TTFM is one of the most widely used methods for intraoperative graft assessment. It works by sending ultrasonic waves across the graft and measuring the time it takes for the signal to pass through, providing real-time data on blood flow [19]. Key parameters measured include Mean Graft Flow (MGF), Pulsatility Index (PI), Diastolic Filling Percentage (%DF), and Backward Flow (%BF) [20]. TTFM is non-invasive, easy to use, and provides immediate feedback on graft function, allowing surgeons to identify and address issues such as flow competition [21,22,23] or anastomotic failure [24] during the surgery. Studies have shown that TTFM reduces the need for postoperative reoperations by detecting issues early [25].

***ii.***  ***High-Resolution Ultrasound (HRUS)***

HRUS offers real-time imaging of the graft and surrounding vasculature, providing detailed information on the structural integrity of the graft, such as the presence of kinks, stenosis, or poor anastomotic techniques [26,27,28,29]. Combining HRUS with TTFM has been shown to increase the diagnostic accuracy of graft assessments. Di Giammarco et al. [28,29] demonstrated that using HRUS to guide the reclassification of flow parameters significantly improved intraoperative decision-making, particularly in cases where graft flow was borderline according to TTFM data. Despite the evidence supporting their efficacy, the adoption of TTFM and HRUS remains limited. Many surgeons are reluctant to integrate these technologies into their practice due to concerns about cost, complexity, and workflow integration [30].

Other methods are used for the same purpose even if less frequently nowadays. We hereafter report them for the sake of completeness. Each of them has advantages and disadvantages; the actual diffusion in the routine practice reflects equipment, operational cost, and potential toxicity more than advantages in diagnostic accuracy, and these issues are in favor of the first two described above. We hereafter report in detail the characteristics of those less diffused methods [13,31].

***iii.***  ***Indocyanine Green (ICG) Angiography***

Indocyanine green angiography (ICG) is a method that involves injecting the fluorescent dye indocyanine green into the bloodstream and using near-infrared fluorescence imaging to visualize blood flow through the grafts [32,33,34]. ICG angiography allows for the real-time assessment of graft patency by visualizing the flow of blood through the coronary arteries and bypass grafts. While less invasive than traditional angiography, ICG angiography may not detect deeper graft issues such as microthrombosis or distal anastomotic stenosis. Furthermore, there have been many reports about allergic reaction, which is probably dose dependent and sometimes lethal [35,36,37]. After a timespan in which the method was almost abandoned, it was reproposed in robotic cardiac surgery [38] even if its use is nowadays prevalent in laparoscopic surgery [39].

***iv.***  ***Intraoperative Conventional Angiography***

Considered the gold standard for assessing graft patency, intraoperative coronary angiography involves injecting contrast dye directly into the grafts and capturing real-time X-ray images to assess flow and detect blockages [40,41,42,43]. Intraoperative coronary angiography provides the highest diagnostic accuracy, with sensitivity rates exceeding 95% [44]. However, the procedure is invasive, requires catheterization, and is associated with higher procedural costs and potential complications either from arterial access or native coronary vessels or from contrast agents and radiation exposure, with the latter being reported less frequently compared to the past [45,46].

***v.***  ***Thermography***

Thermography is a non-invasive technique that detects temperature variations along the graft, which are used to infer the presence of blood flow [40,47]. The underlying principle is that functioning grafts exhibit a steady temperature, while areas with insufficient blood flow show temperature fluctuations [48]. However, thermography is less accurate than other methods like TTFM and intraoperative coronary angiography, with sensitivity ranging from 60% to 70%, limiting its widespread adoption in clinical practice [49,50,51]. It is more of a historically valuable method that is currently infrequently used in clinical practice. We therefore did not consider it for a detailed discussion.

### 3.2. The Question

In spite of the efficacy of all the methods proposed to assess the intraoperative graft patency, from the less to the more invasive and from the cheaper to the more expensive one, an insurmountable reluctance to adopt them in routine clinical practice still remains.

The question we put to AI, namely ChatGPT 4o, is the following: **Why are cardiac surgeons so reluctant to use Transit-Time Flow Measurement (TTFM) and High-Resolution Ultrasound Imaging (HRUS) for verifying intraoperatively coronary grafts?**

In Table 1 we report the reasons listed via GAI.

By refining manually the search, we found in one case a detailed citation of the reasons [51].

From this study, a further technical caveat concerns the usefulness of the skeletonization of the mammary artery with the fear of its damage due to traction during the measurement procedure. Finally, their experience talks in favor of the influence of a number of measurements on the accuracy of prediction: the more you measure, the greater the accuracy.

We considered each point reported in Table 1 as hypotheses to be investigated, as reported hereafter.

## 4. Results

### 4.1. Diagnostic Accuracy of Intraoperative Methods for Coronary Graft Assessment

#### 4.1.1. Transit-Time Flow Measurement (TTFM)

TTFM is a commonly used tool for measuring graft flow in coronary artery bypass grafting (CABG). However, its efficiency can vary, especially in cases involving sequential grafting. According to some authors [52], TTFM metrics such as Mean Graft Flow (MGF) and the Pulsatility Index (PI) may require adjustment for different graft configurations. The same results were published by Drost C, et al. [53]. The study noted that TTFM’s sensitivity if used alone is as low as 25–40%, while its specificity can reach 85–98%. Furthermore, some authors documented good sensitivity and specificity (96.2% and 76.9%) of TTFM alone on arterial grafts, setting up the cutoff values of MGF and PI to 15 mL/min and 2.5, respectively [54]. These discrepancies highlight the importance of interpreting flow metrics carefully, especially in complex cases. A special case is that of the use of sequential grafting and/or composite conduits, (Y- or T-shaped) either with arteries alone or combined with saphenous veins. A prospective trial (registered on the Australian New Zealand Clinical Trial Registry with number ACTRN12622000774729. https://anzctr.org.au/ACTRN12622000774729.aspx (accessed on 30 October 2024)) is currently ongoing in the Czech Republic.

At the moment, it is the first trial concerning the TTFM evaluation on composite conduits. Before the results are available, it should be suggested in these situations to combine TTFM with HRUS to achieve sufficient reliability of the verification.

Talking about the single anastomoses, in a systematic review and metanalysis published in 2019 [55], sensitivity rates (describing the accuracy of TTFM) varied from 25% to 45.7% whereas specificity was between 94.1 and 98.4%. The Negative Predictive Value (NPV) ranged from 71.9% to 98.0%. On the other hand, the Positive Predictive Value varied from 10.0% to 84.0%. These NPV and PPV values were based on the outcomes of angiography performed intraoperatively on the 4th postoperative day.

Recently, in an attempt to speculate on the diagnostic accuracy of TTFM alone, some authors have reported the results of a comparison between TTFM, conventional coronary angiography, Coronary CT-scans, and the Hyper Eye Medical System (HEMS), a colored representation of ICG angiography. They considered the diastolic portion of the flow trace demonstrating that the maximal graft flow acceleration in that phase is a possible predictor of graft failure with a sensitivity of 72.7% and a specificity 80.4% [56].

#### 4.1.2. High-Resolution Ultrasound (HRUS)

The use of ultrasound on coronary circulation was applied for the first time in coronary surgery by the end of the 1980s [24]. When combined with TTFM, HRUS significantly improves the diagnostic accuracy of ultrasound coronary graft assessments. In a study published in 2014 [28], the combination of TTFM and HRUS provided a sensitivity of 85–90% and specificity of 95–100%, with an overall increase in diagnostic accuracy close to 100%. This combination has proven particularly useful for identifying structural problems, such as kinks or stenosis, that are not evident from flow measurements alone. In addition, the same authors published a case series in which the intraoperative diagnostic value of HRUS to detect the presence of significant coronary stenosis in no-angiography patients was demonstrated [57]. This is a demonstration that morphology and function are equally important in the process of graft verification.

Recently, artificial intelligence was investigated in the context of the machine learning technique. The aim is to develop an algorithm to automatically recognize coronary vessels in their course and orientation, before and after anastomosis construction. The final goal of the study was to develop software for intraoperative ultrasound application [58].

#### 4.1.3. Indocyanine Green (ICG) Angiography

A study by Desai et al. (2006) [32] reported sensitivity and specificity values of 83.3 and 100%, respectively, to detect a >50% stenosis compared to the 25% and 98.4% values for TTFM. The development of an evolution of ICG angiography demonstrated even better performance [59,60,61].

#### 4.1.4. Perioperative Conventional Angiography

Compared to TTFM, angiography offers a sensitivity of 95–100% and specificity of 85–95%, though its invasiveness and global cost can limit routine use. Despite these challenges, it is the most accurate method available for detecting graft issues during and after surgery [60]. Abdulla J. et al. reported that CCA showed sensitivity, specificity, and positive predictive and negative predictive values for native coronary arteries of 86% (85–87), 96% (95.5–96.5), 83%, and 96.5% via per-segment analysis; 97.5% (96–99), 91% (87.5–94), 93%, and 96.5% via per-patient analysis; 98.5(96–99.5), 96(93.5–97.5), 92, and 99% for CABGs; 80(70–88.5), 95(92–97), 80, and 95% for stent restenosis; and 87(86.5–88), 96(95.5–96.5), 83.5, and 97% via overall per-segment analysis [62]. Table 2 summarizes the comparative data regarding the diagnostic accuracy of all the described technologies.

### 4.2. Cost and Resource Allocation

As far as the cost effectiveness is concerned, the only available published evidence about a Health Technology Assessment analysis of medical devices is that reported by the NICE (National Institute for Health and Care Excellence) based in UK; the only methods analyzed are TTFM and HRUS. In Table 3, we report some documents filed on the Health Technology Assessment website nice.org.uk (accessed on 30 October 2024).

#### 4.2.1. Transit-Time Flowmetry (TTFM)

TTFM is widely used for its relative affordability compared to other techniques. The only omni-comprehensive evaluation of this issue is reported in NICE publications concerning the use of TTFM alone [63] or combined with high-resolution imaging [64].

#### 4.2.2. High-Resolution Ultrasound (HRUS)

Even though the combination of TTFM and HRUS has been available in a single piece of equipment since 2009, there is little information about the procedural cost, and these data are reported in the last update of the NICE report but are limited to the hardware cost (Table 3), without precise information about the cost per procedure [64]. However, some authors [28,29] showed that HRUS significantly improves graft assessment when combined with TTFM, enhancing diagnostic accuracy to approximately 100%. This combination is considered cost-effective in preventing graft-related complications postoperatively, suggesting that in front of the higher price of the combined hardware, the cost saving is equally higher.

#### 4.2.3. Indocyanine Green (ICG) Angiography

There is a lack of literature data about the procedural costs of ICG angiography. The recommendations from NICE were published in 2004 [65] but the authors did not provide any information about the cost of the procedure. Among the few data available, we collected some information reported below. The cost of indocyanine green (ICG) angiography for coronary graft verification can vary depending on several factors, such as the healthcare provider, location, and the specific setup of the procedure. ICG angiography involves the injection of a fluorescent dye (indocyanine green) and the use of a specialized camera to visualize blood flow and graft patency during coronary artery bypass grafting [34].

In detail:

1. *ICG contrast dye*: the cost of the dye itself is relatively low, often around USD 200 to USD 500 per dose. This can slightly vary based on the country and supplier;
2. *Specialized equipment and personnel*: the procedure also requires specialized imaging systems (e.g., near-infrared fluorescence cameras) and trained personnel to carry out the procedure. The costs for using this equipment in a hospital setting can add several thousand dollars to the overall procedure, depending on the hospital or surgical center;
3. *Overall procedure cost*: when the use of ICG angiography is set as part of CABG surgery, the additional cost may range from USD 1000 to USD 3000. This is an approximation and can vary based on the healthcare facility, region, and specific case complexity (the more anastomoses, the higher the cost) [66,67,68,69].

Another important aspect to be considered is the amount of drug wasted each case which contributes to the total cost of the procedure as reported in Table 4 [70].

#### 4.2.4. Intraoperative Conventional Angiography

The cost per procedure for conventional contrast angiography depends on several factors, including hospital or clinic location, country, and whether it is conducted in a public or private setting.

In Table 5, the cost breakdown for conventional contrast angiography is reported [71].

In Table 6, the overall estimated cost per procedure in different countries is reported. The estimate is different according to location, especially if US and Europe are compared, being lower in the latter region.

### 4.3. Procedural Costs and Cost-Effectiveness of the Procedure

The adoption of intraoperative graft assessment technologies like Transit-Time Flow Measurement (TTFM) and High-Resolution Ultrasound (HRUS) in coronary artery bypass grafting (CABG) brings significant upfront costs, but the potential long-term savings make these tools a viable investment for hospitals seeking to improve patient outcomes. Understanding the financial implications of these technologies is key to addressing the reluctance among cardiac surgeons and healthcare institutions to incorporate them into routine practice.

#### Upfront Costs of TTFM and HRUS

TTFM devices typically cost between USD 50,000 and USD 100,000, depending on the manufacturer and the specific features of the system. This includes the hardware required to generate and interpret flow measurements, as well as the necessary software for data analysis. The HRUS system increases the total cost of the equipment by almost EUR 50,000. These costs are significant, especially for smaller hospitals or those operating in low- and middle-income countries where financial constraints are a major concern [64].

Beyond the purchase of the equipment, there are additional costs associated with training personnel to use these technologies effectively. Surgeons, technicians, and operating room staff require specialized training to perform and interpret TTFM and HRUS data accurately, which can cost anywhere from USD 10,000 to USD 20,000 per team. These training costs can be a deterrent, especially in regions where budgets for healthcare staff education are limited. Moreover, maintenance and service agreements for these devices must be factored into the cost equation. Annual maintenance fees can range from USD 5000 to USD 8000 depending on the complexity of the system and the service contract with the equipment provider. These recurring costs add to the financial burden for hospitals that are already struggling with tight operating budgets.

### 4.4. Cost Savings in Reducing Complications

Despite the substantial upfront costs, the long-term savings achieved by reducing postoperative complications and the need for reinterventions can make the use of TTFM and HRUS highly cost-effective. The REQUEST Study [72] demonstrated that using TTFM and HRUS to verify graft patency intraoperatively reduced the incidence of graft-related complications such as anastomotic failure and flow competition. These technologies allowed surgeons to identify problematic grafts during surgery, enabling immediate corrections that would otherwise have required an early reoperation.

The cost of a reoperation for a failed graft is estimated to be around USD 30,000 to USD 50,000, depending on the complexity of the procedure and the associated hospital stay. Moreover, patients requiring reoperations often have longer hospital stays, which further increases costs. By reducing the need for reoperations, TTFM and HRUS can lead to significant savings, offsetting the initial investment in the technology within a few years of implementation.

Intraoperative Conventional Angiography, while providing the highest diagnostic accuracy, also carries high costs. The equipment for coronary angiography is present in all the hospitals in which CABG is performed; so, even if the cost of the equipment does not need to be calculated, the cost of the specialized staff including interventional cardiologists and technicians trained in using fluoroscopy equipment dedicated to this exam should be added as well as the cost of all the disposable equipment needed [43]. Finally, intraoperative coronary angiography exposes patients to additional risks, such as contrast-induced nephropathy and radiation exposure, which can lead to further complications and increase hospital costs.

### 4.5. Complexity and Workflow Integration

Integrating a graft verification procedure into the surgical workflow can be challenging, particularly in high-volume centers. Many surgeons are concerned that the use of these tools will extend operating times and disrupt established routines. If it is true for TTFM and HRUS, it is even more true for other methods of intraoperative evaluation of coronary grafts. We then analyzed the increase in surgical times for all methods of controlling the patency of grafts. Cardiac surgeries, especially coronary artery bypass grafting (CABG), are time-sensitive procedures, and any additional step that prolongs the operation may be met with resistance. This is particularly important in high-volume centers where efficiency is critical, and operating room time is a premium resource. Table 7 summarizes the time to that will be added to surgical procedures for each technology.

#### 4.5.1. TTFM (Transit-Time Flow Measurement)

TTFM is by far the most commonly used intraoperative method due to its ease of use and relatively low cost. However, the integration of TTFM into the surgical process can still add 10–15 min to the overall procedure. The time increase is due to the need to place flow probes, take multiple measurements, and interpret the results at each graft site. While TTFM offers valuable data on graft flow, concerns remain that it may disrupt the rhythm of the surgery, particularly when multiple grafts are involved.

#### 4.5.2. HRUS (High-Resolution Ultrasound)

While offering excellent anatomical details and the ability to identify structural issues like intimal flaps or subcritical stenosis or dissection, HRUS adds even more time to the procedure. Studies show that it typically extends the OR time by 15–20 min. The need to acquire high-resolution images and interpret the data in real time requires coordination and sharing objectives in the surgical team. In addition, setting up the ultrasound device and performing the imaging requires more logistical effort compared to TTFM. This extra time and complexity contribute to surgeon reluctance, as it may disrupt the normal flow of the operation.

#### 4.5.3. Indocyanine Green (ICG) Angiography

ICG angiography is a valuable tool for real-time visualization of graft patency and has been shown to have high diagnostic accuracy. However, it also increases the overall surgery duration, primarily due to the time required for setting up the camera, diluting the fluorescent dye and administering it; ultimately, a bit more time is needed for interpreting the results. From administering the dye to image interpretation, it is reported that a time interval of 3 min/graft is added [74]. It could be calculated for a standard triple CABG that a total increase in OR time varies from 20 to 30 min per surgical procedure. Moreover, ICG is less portable and more resource-intensive; in the case of simultaneous procedures in different operating rooms, the time needed rises if compared to TTFM and HRUS. These factors make ICG more disruptive, particularly in centers focused on minimizing OR time.

#### 4.5.4. Intraoperative Conventional Angiography (CA)

It is the gold standard for graft assessment, providing the most detailed information about graft patency. However, it is also the most time-consuming and invasive option and the most expensive as well. Conventional angiography can add at least 30–45 min to the procedure due to the need for catheter placement, contrast injection, and imaging. This is in the case that surgeons work in a hybrid room. The time is longer if the patient needs to be moved to an adjacent catheterization lab room. The risks associated with contrast agent infusion, the possible bleeding from the puncture site as the patient is still on residual heparine, and the overall complexity of the procedure further contribute to surgeon reluctance. While it offers the highest diagnostic accuracy, the significant time and logistical burden make it less appealing for routine use, particularly in lower-risk cases.

#### 4.5.5. Balancing Workflow Disruption with Clinical Benefits

While each of these methods adds time to the surgery, they offer different levels of diagnostic accuracy and benefits. For instance, TTFM, while adding the least time, offers lower sensitivity compared to HRUS and ICG angiography. On the other hand, intraoperative standard angiography provides the highest accuracy but significantly increased OR time, making it suitable for high-risk cases where absolute certainty is needed. Surgeons must balance these factors when choosing which technology to integrate into the workflow. While the fear of disrupting normal surgical rhythm is valid, the potential benefits in preventing postoperative complications, reducing the need for reoperations, and improving long-term outcomes can outweigh the additional time required during the surgery.

### 4.6. Training Requirement

The successful integration of Transit-Time Flow Measurement (TTFM), High-Resolution Ultrasound (HRUS), and indocyanine green (ICG) angiography into intraoperative coronary graft assessment requires adequate training for surgeons and the entire surgical team. The complexity of these methods, their interpretation, and their impact on surgical workflow necessitates specific expertise.

#### 4.6.1. TTFM

TTFM is relatively straightforward in its operation but still requires training to ensure accurate setup, use, and interpretation of the data. There are two types of issues to be considered in order to best manage the flow detection procedure in vascular grafts. The first is a need for basic knowledge of coronary physiology and pathophysiology which, if it is obviously possessed by the surgeon, must be implemented into nurses’ knowledge and technicians who participate in operating theatre activity; the second concerns knowledge of the device through which flows and other parameters useful for diagnosis are detected. If we consider the almost rudimentary conditions of the beginnings of the introduction of the method in the late 1990s, it must be noted that the current equipment allows parameters to be detected and recorded very easily. In addition, the recent implementation of options in the management database makes these devices capable of rational and quick data mining, even for the purposes of scientific analysis.

#### 4.6.2. HRUS

HRUS is a more complex modality than TTFM, requiring specialized training in ultrasound technology and real-time imaging interpretation. The learning curve is steeper and should adhere to the following principles stated by the European Association of Echocardiography [73]. In Table 8, we report the suggested steps to reach knowledge for competence in echocardiography that are definitely applicable to the present discussion.

Beyond specifical knowledge in ultrasound physics, other skills are required to be fulfilled by the whole team in different procedural steps, either during flowmetry alone or combined to ultrasound imaging:

- Probe placement: correct placement of the flow probe is essential to obtain accurate measurements. Surgeons must learn how to place the probe on grafts without causing mechanical disturbances;
- Interpretation of flow data: understanding key TTFM parameters like Mean Graft Flow (MGF), the Pulsatility Index (PI), and Percent Backflow (%BF) is crucial for determining graft patency. Misinterpretation of these metrics can lead to unnecessary graft revisions or missed complications;
- Image interpretation: HRUS images provide real-time feedback during surgery, but interpreting those images requires experience and training to avoid false positives or negatives;
- Device and probes correct maintenance as far as sterilization is concerned.
- Training time: typically, surgeons and surgical assistants can become proficient in TTFM within several hours of hands-on training, often provided by device manufacturers such as Transonic Systems. However, ongoing experience is required to refine the interpretation of complex cases. We recently registered a trial (https://clinicaltrials.gov/study/NCT06589323?firstPost=2012-03-03_#study-plan (accessed on 31 October 2024)) [75] in the aim to train young resident surgeons for a complete implementation of the method in daily practice.

#### 4.6.3. ICG Angiography

ICG angiography, while offering excellent real-time visualization of graft flow, also requires substantial training for both administration and image interpretation. The training requirements include the following:

- Dye dilution and administration: ICG dye must be injected at the right moment, requiring coordination with the surgical procedure. Surgeons and anesthesiologists need to be trained in the precise timing and dosage of the dye;
- Fluorescence imaging equipment: operating the fluorescence imaging system involves training on how to visualize grafts under infrared light. Technicians or support staff must handle this equipment, ensuring that the images are captured at the right time;
- Interpreting fluorescent images: ICG angiography provides dynamic, real-time visualization of graft flow. Surgeons must be able to differentiate between well-perfused grafts and areas of poor flow based on the fluorescence patterns;
- Training time: proficiency in ICG angiography generally takes weeks to months of training, particularly for staff operating the imaging equipment. Surgeons also need time to become familiar with the interpretation of the angiographic images to ensure accurate assessment of graft patency.

Regarding the estimate of the duration of training, no data in the literature are reported; moreover, rather than calculating this requirement in terms of time, it is better to express it by counting the number of clinical cases required. In the trial we devised and registered [75], concerning the development of the ability to manage the echo probe, we made use of the scanning of the radial artery which, being particularly superficial, can simulate the scanning of a coronary artery. Each participant, therefore, has the opportunity to practice outside the operating theatre, shortening performance times. The results of the LEARNERS trial will be available in 2025.

In Table 9, we tried to hypothesize the training duration based on personal clinical experience, direct or indirect as in the case of conventional angiography.

### 4.7. Skepticism Regarding Clinical Utility

Focusing the attention of the readers on the most diffused methods in comprehensive graft verification, we report some of the most representative studies on TTFM and HRUS. With respect to conventional angiography, the fundamental question concerning all the methods is whether technology is better than visual inspection and finger palpation.

The first observational study which demonstrated the clinical utility of TTFM was published by Beçit and Coll. [76]. They analyzed two groups of patients revascularized with on-pump CABG, 100 patients each were operated on across the introduction of Transit-Time Flow Measurement. In the group submitted to the TTFM control, whose data were prospectively collected, a significant reduction in negative events including myocardial infarction, need for IABP, and death was documented. It should be noted that the use of TTFM in myocardial revascularization under CPB and an arrested heart [76] resulted from that time of paramount importance because the method had been introduced ten years earlier for the validation of coronary surgery on a beating heart.

An analysis on 1829 patients submitted to an angiographic control included in the PREVENT IV trial protocol [77] aimed to assess the clinical outcome of saphenous vein graft failure showed an increased rate of reinterventions in the case of SVG disease even if no difference was found about death and myocardial infarction rate. The first 2400 patients (out of 3014 enrolled in total) were assigned to the planned angiography candidate group, 12 to 18 months after coronary bypass surgery. A total of 571 patients were not controlled, as 91 died and 480 did not return for follow-up. Although mortality was not directly dependent on venous graft disease but rather on reintervention, sometimes urgently needed for clinical instability, the authors document an indirect dependence on venous graft failure; in addition, they report a 0.9% composite end point of death or new myocardial infarction in patients with patent SVGs compared with 13.9% for patients with at least one occluded SVG. There is also an underestimation of the effect due to the number of patients excluded from angiography because they had died in the meantime or refused to undergo the examination. Finally, the importance of internal mammary artery patency undoubtedly influencing survival was not considered. The authors concluded advocating any strategy able to reduce SVG failure and its effects on clinical outcome.

In the GRIIP trial [78], two randomized groups were compared: the Imaging Group (intraoperative graft assessment using fluorescence angiography (Novadaq SPY ICG angiography system; Novadaq Technologies, Toronto, Ontario, Canada) and TTF (Medtronic MediStim flowmeter, Medtronic, Inc, Newport, Calif), with a strategy of graft revision based on a priori *criteria*, and the Control Group according to criteria reported in the study. It is noteworthy that of 156 patients randomized in the two groups with 467 grafts tested, 76% were controlled with ICG and TTFM and only 7 (1.7%) were controlled with TTFM alone.

The most important finding reported in the study is that in the Imaging Group, the authors document that eight grafts (3.6%) met the criteria for revision even though only four grafts (1.7%) were revised; conversely, no grafts were found in the Control Group that met the criteria for revision, and despite this result, five grafts (2.1%) were, however, revised. Although the data do not appear to be of unambiguous interpretation, they testify the inaccuracy of the criteria followed in the Control Group and, on the other hand, the skepticism in following the indications of the imaging examinations, demonstrating in both cases the decision-making bias of the surgeon.

Another study to be discussed is the ROOBY Trial whose original design was published in 2007 [79]; it is a randomized study concerning a comparison between on and off-pump CABG procedures. As the authors wrote in the protocol, it showed some declared limitations. The areas of concern are the experience of surgeons in off-pump procedures, the participation of residents in the study, and the value of the precise neurocognitive tests that were performed. The study was conducted from 2002 to 2008 at 18 Veteran Affairs Medical Centers. TTFM was introduced into the trial in July 2003. The endpoints were 30-day and 1-year complications as death, myocardial infarction (MI), new revascularization, and 1-year angiographic patency; for the ROOBY-FS Trial [80], the same clinical outcomes were reported at 5 years after CABG. The FS study compares angiographic results and the clinical outcome of 1067 patients who underwent TTFM evaluation to 501 patients not assessed with this method. It is noteworthy that TTFM was conducted in patients with significantly smaller caliber coronaries than the uncontrolled patients and of worse wall quality; in addition, the control group patients had undergone significantly more anastomoses and sequential or Y-conduit grafts. Finally, the TTFM-controlled patients had undergone a significantly increased number of intraoperative graft revisions. These findings documented the propensity to adopt TTFM graft verification in difficult situations even if not declared in the study protocol.

Kieser et al. [81], in 2010, reported on a series of 1000 arterial grafts (LIMA to LAD) in 336 consecutive patients demonstrating the importance of a PI value > 5 as a predictive parameter of worse clinical outcome even if the other parameters are normal. On the basis of this study, TTFM was endorsed for the first time in the European Guidelines of Coronary revascularization [82].

Herman et al. [83] considered the outcomes of patients submitted to TTFM verification. They report those showing a normal PI (<5) in all grafts compared to those who had a PI > 5 in at least one graft (19% of the total reported population). The in-hospital composite outcomes of adverse cardiac events (death, MI, revascularization, prolonged ventilation, and low cardiac output syndrome) were significantly higher in patients with an abnormal PI (31% versus 17%; OR, 1.7 [95% CI, 1.1–2.7]; *p* < 0.0001).

A propensity score analysis published in 2022 by Laali et al. [51] on 910 patients divided into two groups (430 submitted to TTFM; 480 without any control) considering as the primary endpoint the occurrence of in-hospital MACE (in-hospital mortality, perioperative myocardial infarction, cardiac arrest, need for intra-aortic ballon pumping or ECMO support, need for urgent postoperative coronary angiography, and postoperative re-procedure or PCI). They report a significantly lower incidence of MACE in the TTFM group (3.3% vs. 6.9% *p* = 0.014) demonstrating at crude regression the protecting role of the method (OR:0.46; CI: 0.23–0.85, *p* = 0.016).

Finally, the REQUEST study [72] demonstrated the efficacy of TTFM and HRUS in reducing postoperative complications. The analysis involved seven centers worldwide and 1046 patients submitted to CABG intraoperatively controlled using TTFM and HRUS imaging. In 25% of cases, a change in the surgical planned procedure was decided on the basis of aorta (9.9%), in situ conduits (2.7%), and coronary target (22.7%) problems. Graft revision occurred in 7.8% of cases, including revisions of the proximal and/or distal anastomosis in 6.6%. In-hospital adverse event rates were 0.6% for mortality, 1.0% for cerebrovascular events, and 0.3% for myocardial infarction. Even if more long-term evidence and prospective randomized data are needed, those that are available are definitively more convincing compared to what was reported in the NICE analysis regarding TTFM [63] and high-resolution imaging [64].

### 4.8. Worldwide Adoption of TTFM and HRUS and Other Technologies

We tried to reach precise data about the rate of adoption of all the methods for intraoperative graft verification, but unfortunately, they are not verified. The data reported here could be defined just as a rough estimate and should be analyzed with criticism considering the different percentage of systems bought by customers versus their actual usage, the latter being definitely lower. Adoption of TTFM and HRUS varies significantly by region. In North America and Europe, more than 70% of cardiac centers have integrated TTFM into their practices, while HRUS is used in about 30% of centers. In contrast, adoption rates in Asia and developing regions remain low due to financial and infrastructural barriers.

Data should be considered as reliable in the case of TTFM and HRUS as those two technologies are prioritized in the field of cardiovascular surgery (cardiac or vascular). As far as ICG is concerned, the presence of this technology in a hospital is not exclusive for cardiovascular use being rather oriented nowadays towards general surgery.

Geographical distribution of TTFM w/wo HRUS

The geographical distribution of the use of procedures for coronary bypass verification using Transit-Time Flow Measurement (TTFM) and High-Frequency Ultrasound Imaging (HFUS) reveals key insights. MediStim (Oslo, Norway www.medistim.com (accessed on 30 October 2024)), a leading provider in this area, reports the following information [84] (Table 10):

Very unfortunately, we did not find corresponding data about the other TTFM equipment produced by Transonic Inc. (Ithaca, NY, USA).

## 5. Discussion

This literature review performed with the partial support of AI has allowed us to grasp what we could call “the humors” related to the use of intraoperative procedures for the verification of coronary grafts by surgeons. The moods are certainly not so-called scientific data; in addition, they are sometimes hidden between the lines of the publication and, therefore, are not easy to highlight.

The main objective of the verification, however, does not seem to be the evaluation of the patency of the grafts but the proof that a certain technological option may affect the result of the verification. In fact, considering the costs of managing the immediate complications of a failed myocardial revascularization and the possible management of short-to-medium-term graft occlusion, even the more expensive control method appears advantageous.

Moreover, in this review, very expensive and very invasive methods have been compared with less expensive and very non-invasive ones. On the other hand, talking about diagnostic accuracy, it would be correct to introduce a further distinction, namely that between isolated and combinable methods.

Seen from this aspect, it is justified to have emphasized the considerations about TTFM and HRUS which, if taken in combination, fill the gap in diagnostic accuracy.

So far, the arguments concern the surgeon’s choice.

Considering the situation from the patient’s point of view in a holistic and ethical vision of medicine, the answer to the question he might ask leaving the operating theatre ‘can I know if my coronary grafts are working?’ should be a certain assurance of the effectiveness and efficiency of the intervention performed, just as the request for minimal invasiveness of the surgical access is answered visually by the length of the skin incision. Minimal invasiveness in coronary surgery should, therefore, involve the functional aspect of the intervention that minimizes the need of a short-term repeated procedure.

It is, therefore, not wise to continue to advocate the use of the most invasive, most expensive, and riskiest method just because the less expensive ones have a diagnostic accuracy of a few percentage points less than the others. The diagnosis comes from the combination of clinical acumen, technical ability, intellectual honesty (the surgeon always knows if it is likely that there have been errors along the way), and help from the diagnostic method that is more guaranteed than those provided.

All the reasons that have been read and that have been reported in this review of the literature can be defined as a “procedural outline”:

(1) The training of surgeons and operating room staff is a usual procedure that is adopted every time a new device is introduced and that in some cases (such as robotic surgery) is much more complex and expensive than that needed to consolidate a procedure such as the evaluation of a coronary graft function;
(2) The cost per procedure (calculated as inclusive of the cost of the device) must be assessed in relation to the number of bypass procedures performed by the center. It goes without saying that there is a minimum number of coronary surgeries to be considered in the calculation, and this coincides with the limit of 200/year below which the presence of a cardiac surgery department is not justified in many health organizations;
(3) The clinical benefits of graft control have been highlighted in many studies and for many years. Some complain about the lack of prospective randomized trials or studies in which a comparator is present [64]. Admitting a comparator (in this case, a group of patients undergoing the same surgery but without the aid of intraoperative control) means reasoning according to purely statistical aspects and not according to the principles of personalized medicine; in this way, some patients would be removed from the reduction in the risk of complications in an evidently unethical choice. Finally, the results of multicenter studies in which intraoperative control was routine [72] demonstrate the evident reduction in post-surgical complications: the rate of perioperative myocardial necrosis was reduced close to zero, and the overwhelming reduction in cerebrovascular accidents was reduced by 6 to 2%.

The results achieved with the use of TTFM and HFUS are undoubtedly the same as those achieved following intraoperative angiography with indocyanine green injection or conventional contrast dye. What changes is the risk of toxicity and allergic reactions to the contrast medium which is a corollary to a higher procedural cost than the ultrasonographic method in the face of diagnostic accuracy parameters of slightly higher or at least equal values if we compare the combined use of Transit-Time Flowmetry and high-resolution imaging, which compensates for the low sensitivity of flowmetry alone. As for the increase in surgical times resulting from the use of control methods, it should be emphasized that TTFM and HRUS are the ones that result in the smallest increase in this parameter.

Quality control in cardiac surgery deserves further consideration. A few decades ago, the intraoperative use of Transesophageal Echo (TEE) was proposed to check the outcome of mitral valve repair. Although the presence of a residual mitral regurgitation is not connected to immediate adverse clinical outcomes, it would impair prognosis, and today, no valve surgeon would leave the operating room without having seen the full success of the repair on the echo screen. In coronary surgery, the intraoperative control can be equally crucial as it could deeply contribute to the reduction in short- and mid-term events; this should be a convincing reason for this procedure to become routine.

## 6. Conclusions

Two important concepts can be deduced from this study. The first is about the method of research partially based on the use of AI controlled and to some extent validated by human intelligence, experience, and sensitivity, which are still indispensable in such contexts. The aid of tools capable of performing controlled searches in a few seconds is undoubtedly useful for the considerable time saving in completing scientific research. On the other hand, plagiarism and hallucination are two pitfalls of GAI. Therefore, it should be mandatory to consider the published opinions on this matter [1,85,86].

The second concept regards the merit of this research, whose results constitute a useful addition to confirm that ultrasound is the winning technology in intraoperative coronary graft testing. In more detail, the graft functional verification by means of TTFM combined with the morphological verification via HFUS guarantees the best diagnostic accuracy in less than 10 min together with the best sustainability for the health service systems in both the economic and organizational pathways.

## Figures and Tables

**Table 1:** Common issues limiting the extensive adoption of intraoperative graft verification procedures.

Diagnostic accuracy
2. Cost and resource allocation
3. Procedural cost and cost effectiveness
4. Cost savings in reducing complications
5. Complexity and workflow integration
6. Training requirement
7. Skepticism regarding clinical utility
8. Worldwide diffusion and credit

**Table 2:** Diagnostic accuracy of the most diffused methods for intraoperative graft verification.

Method	Sensitivity (%)	Specificity (%)	PPV (%)	NPV (%)	Reference
TTFM	25–45.7	94.1–98.4	10–84	71.9–98	Thuijs et al. (2019), [55]
HRUS + TTFM	85–90	95–100	85–100	85–100	Di Giammarco et al. (2014), [28]
ICG Angiography	83–100	85–100	85–100	85–100	Desai et al. (2006), [32]
Intraoperative Coronary Angiography	95–100	85–95	80–100	90–100	Abdalla et al. (2007), [62]

TTFM = Transit-Time Flow Measurement, HRUS = High Resolution UltraSound, ICG = Indocyanine Geen Angiography, PPV = Positive Predictive Value, NPV = Negative Predictive Value.

**Table 3:** Procedural costs of TTFM use (w/wo HFUS) with respect to the clinical evaluation (simplified from NICE reports).

	NICE rev. 2011 (TTFM Alone) [63]	NICE rev. 2023 (TTFM + HFUS) [64]
Equipment cost	Based on VeriQ console price: GBP 32,000 (anticipated lifetime 10-y)	MiraQ console GBP 81,550
Probe cost (1.7 probes per procedure)	GBP 1582 (for 30 times use)	Flow probe: GBP 1720 L15 Imaging probe: GBP 8715
Cost per patient (included equipment) based on MiraQ console cost/220 days of usage per year, **annual maintenance cost** included equal to GBP 1800/year payable 2nd year on)	GBP 111	N/A
Time added to surgical procedure for 3-CABG	2.35 min	N/A
revision rate	6.6% (minor 2.3%; major 4.3%)	N/A (it could be hypothesized that it should lower up to <1% [28]
Cost of the time taken for revision	GBP 11 for minor GBP 180 for major	N/A
Cost of postoperative complications	GBP 1667	N/A
IABP cost	GBP 2657 per episode (1% in TTFM group vs. 7% in clinical assessed group)	N/A
Cost saving (in case of TTFM use compared to clinical assessment)	Maximum cost saving for TTFM use GBP 323/patient (0% IABP) Minimum cost saving for TTFM use GBP 38 (7% IABP)	N/A

**Table 4:** Balance of ICG: injected/wasted.

Typical ICG Use and Waste
A standard vial of ICG typically contains 25 mg of the dye.
For coronary graft verification, around 1 to 2 mg per injection may be used.
Depending on the number of injections (usually 1 to 3 injections per surgery), a fraction of the vial may remain unused.
Estimated Waste:
If a 25 mg vial is used and only 2 to 6 mg are needed for a procedure, approximately 19 to 23 mg of ICG is wasted.
Cost of Waste:
Given that a vial of ICG costs around USD 200 to USD 500, and around 75% to 90% of the dye may go unused, the money loss could range from USD 150 to USD 450 per procedure.

**Table 5:** Cost breakdown for conventional angiography.

Contrast Dye:
○ The contrast dye used in angiography typically costs USD 50 to USD 200 per procedure, depending on the brand and type of contrast agent used.
2. Equipment and Facility Fees:
○ The use of a specialized catheterization lab and angiography imaging equipment is a significant part of the cost. This can range from USD 1000 to USD 5000, depending on the country, hospital, and whether the procedure is outpatient or part of a more extensive surgical process.
3. Radiologist and Surgeon Fees:
○ Professional fees for the cardiologist or radiologist interpreting the angiograms, plus any surgical oversight, can range from USD 500 to USD 2000 or more.
4. Additional Costs:
○ Other expenses, such as intravenous access setup, patient monitoring, and other hospital services, may add USD 200 to USD 1000 to the total cost.

**Table 6:** Cost per patient of conventional coronary angiography compared to coronary magnetic resonance.

Unit Costs	Outpatient in Germany (€)	Inpatient in Germany (€)	Outpatient in the United Kingdom (£)	Inpatient in the United Kingdom (£)	Outpatient in Switzerland (CHF)	Inpatient in Switzerland (CHF)	Outpatient in the United States (US$)	Inpatient in the United States (US$)
CA	588	1207	1055	1934	2580	4638	874	2652
CMR	164 *	393 **	558		1420	/	740	/
SEcho	94	/	213		447	/	303	/
CT	165	/	111		494	/	446	/
SPECT	275	/	406		2183	/	570	/

* cost for a thoracic MR examination, ** cost for MR as a pre-inpatient examination, CA = coronary angiography; CMR = cardiac magnetic resonance; SEcho = standard echocardiography; CT = computed tomography; SPECT = single photon emission computed tomography.

**Table 7:** Time to be added to surgical procedure for each method.

Method	Time to be Added to Operating Time for Each Measurement	Complexity Level	Key Workflow Disruptions
TTFM (Transit-Time Flow Measurement)	<5 min (for a 3 vessels CABG)	Low to Moderate (Training is needed)	Placement of flow probe
			Multiple measurements and data interpretation
HRUS (High-Resolution Ultrasound)	10 min (for a 3 vessels CAGB)	Moderate to High	Real-time imaging setup
			Image interpretation
ICG Angiography	20–25 min [4,66,73]	Moderate	Equipment setup for fluorescence imaging
			Fluorescent dye preparation
			Dye injection and image interpretation
Intraoperative Coronary Angiography	>30–45 min	High	Catheter placement
			Contrast injection
			Time-consuming setup for imaging

**Table 8:** Basic knowledge for competence in echocardiography to be applied to HRUS.

Ultrasound physics
Principles of echocardiographic image formation
Machine settings and instrumentation handling
Normal cardiovascular anatomy, including possible normal variants
Pathological changes in cardiovascular anatomy in different disease states
Normal cardiovascular physiology and fluid dynamics of normal blood flow
Pathological changes in blood flow in different disease states
Potential complications (e.g., for TEE, stress echo, and contrast procedures)

**Table 9:** Minimal training duration for each procedure.

Method	Training Time	Key Training Areas
TTFM (Transit-Time Flow Measurement)	4 to 5 cases (whole team initial training)	Proper probe placement
		Interpretation of flow metrics (MGF, PI, and %BF)
		Operating the equipment
HRUS (High-Resolution Ultrasound)	15 cases (initial training)	Probe handling and image acquisition
		Anatomical knowledge of coronary arteries
		Real-time image interpretation
ICG Angiography	15 cases (initial training)	Dye administration and timing
		Operation of fluorescence imaging equipment
		Image interpretation for graft assessment
Intraoperative Conventional Angiography	20 cases (overall training) Indirect estimate from cardiologists	Catheter placement
		Use of contrast agents
		Detailed imaging interpretation and troubleshooting

**Table 10:** Geographical distribution of graft verification in coronary surgery [84].

Geographical Area	Market Penetration	Actual Usage in Clinical Practice
United States	MediStim’s products account for nearly 30% of the global market	Less than 35% of coronary artery bypass grafting (CABG) procedures (equal to approximately 200,000 cases) are checked using these systems, indicating significant growth potential. Approximately 650 systems are installed across the US.
Canada	About 39% of the global market	About 18,000 CABG procedures are performed annually in Canada, with MediStim technology supporting around 37% of these.
Europe	No information about the global market	Same prevalence as US, globally speaking, from 80% of total CABGs in Germany to 10% in UK.
BRICS Countries (Brasil, Russia, India, China, South Africa)	70% MediStim support	42,000/60,000 CABGs/year verified
Japan and India	Japan high market penetration India 5% market penetration	Japan > 90% total CABGs performed verified India no data

## Data Availability

The data underlying this article will be shared upon reasonable request to the corresponding author.
